# Probe-target hybridization depends on spatial uniformity of initial concentration condition across large-format chips

**DOI:** 10.1038/s41598-020-65563-3

**Published:** 2020-05-29

**Authors:** Alisha Geldert, Haiyan Huang, Amy E. Herr

**Affiliations:** 1 UC Berkeley – UCSF Graduate Program in Bioengineering, Berkeley, United States; 2 Department of Statistics, University of California Berkeley, Berkeley, California 94720 United States; 3 Center for Computational Biology, University of California Berkeley, Berkeley, California 94720 United States; 4 Department of Bioengineering, University of California Berkeley, Berkeley, California 94720 United States

**Keywords:** Bioanalytical chemistry, Biomedical engineering

## Abstract

Diverse assays spanning from immunohistochemistry (IHC), to microarrays (protein, DNA), to high-throughput screens rely on probe-target hybridization to detect analytes. These large-format ‘chips’ array numerous hybridization sites across centimeter-scale areas. However, the reactions are prone to intra-assay spatial variation in hybridization efficiency. The mechanism of spatial bias in hybridization efficiency is poorly understood, particularly in IHC and in-gel immunoassays, where immobilized targets are heterogeneously distributed throughout a tissue or hydrogel network. In these systems, antibody probe hybridization to a target protein antigen depends on the interplay of dilution, thermodynamic partitioning, diffusion, and reaction. Here, we investigate parameters governing antibody probe transport and reaction (i.e., immunoprobing) in a large-format hydrogel immunoassay. Using transport and bimolecular binding theory, we identify a regime in which immunoprobing efficiency (η) is sensitive to the local concentration of applied antibody probe solution, despite the antibody probe being in excess compared to antigen. Sandwiching antibody probe solution against the hydrogel surface yields spatially nonuniform dilution. Using photopatterned fluorescent protein targets and a single-cell immunoassay, we identify regimes in which nonuniformly distributed antibody probe solution causes intra-assay variation in background and η. Understanding the physicochemical factors affecting probe-target hybridization reduces technical variation in large-format chips, improving measurement precision.

## Introduction

Probe-target hybridization over centimeter length scales underpins diverse workhorse assays, including DNA and protein microarrays, immunohistochemistry (IHC), *in situ* hybridization (ISH), and in-gel immunoassays. In such large-format chips, fluorescently labeled probes or targets bind to species immobilized across an area approximating a microscope slide in size (~25 mm × ~75 mm). Large-format chips facilitate either concurrent measurement of 100s to 1000s of samples arrayed as spots, or study of the tissue microenvironment over centimeter distances. Although the large format increases throughput via concurrent measurements, intra-assay spatial variability is often observed, which increases measurement error^1–4^.

The mechanism of spatial bias in probe-target reactions in large-format chips is platform-dependent. When immobilized probes are incubated with a solution containing limited amounts of targets (e.g., DNA microarrays), spatial variation is attributable to diffusive transport limitations and target depletion^1^. In contrast, in other assays (e.g., reverse phase protein arrays, IHC, ISH, and single-cell immunoblots) immobilized targets are incubated with a more concentrated probe solution. The mechanism of spatial technical variation in these immobilized-target, probe-in-excess formats is poorly understood. Hypothesized mechanisms of spatial bias in probe-target hybridization include intra-assay variation in substrate density and permeability^3^ as well as nonuniform reagent distribution due to warped coverslips or evaporation near the edges of the fluid layer^5^; however, few studies have validated or addressed the mechanism of spatial bias. While strategies to reduce spatial bias using internal standards^6^, normalization^3,4^, and other post-processing approaches have been developed – particularly for arrayed systems – these approaches can be challenging to integrate in all assay formats. Understanding the mechanism of spatial variation in probe-target hybridization is crucial to eliminate the root cause of intra-assay technical variation in immobilized-target, probe-in-excess assays.

The amount and mechanism of spatial variability in IHC and in-gel immunoassays (e.g., single-cell immunoblotting^7^) is especially unclear, as complex phenomena impact probe-target binding in these assays. In both IHC and in-gel immunoassays, the target antigen is distributed throughout a sample matrix (e.g., tissue slice or hydrogel) with non-negligible thickness (~10s of µm), rather than being printed on a planar substrate as in microarrays. Local antibody probe concentration within the sample matrix may vary both depth-wise and laterally. Thermodynamic partitioning^8,9^, unknown diffusive timescales into tissue^10^, and variable tissue permeability^11^ reduce probe concentration in the sample matrix and may add variability to Z-directional probe penetration in tissue sections. The fluid layer on a hydrated hydrogel surface or rinsed IHC tissue slice increases variation in the degree of probe dilution^12^. To minimize technical variation due to probe depletion, probe concentrations should be in excess of target^13^; thus, probe concentration must be especially high to overcome thermodynamic partitioning and dilution effects. The necessary high concentration of probe increases the importance of minimizing probe volume to conserve reagents and cost. However, unlike in microarrays, the location of target molecules in tissue sections and single-cell immunoblot chips is unknown; thus, probe must be distributed across the entire surface of the chip and cannot be precision-spotted at defined locations. Additionally, both IHC and single-cell immunoblotting (as well as other immunoassays) rely on antibodies as probes, which exhibit a wide range of binding affinities (probe-to-probe, and lot-to-lot for the same probe)^14–18^. Overall, the complex and variable interplay of thermodynamic partitioning effects, nonuniform probe dilution, and concentration-dependent reaction phenomena raise important considerations for making semi-quantitative protein measurements across large-format chips.

Here, we characterize antibody probe uniformity across centimeter distances in an in-gel immunoassay and determine the impact of initially nonuniform probe concentration on immunoprobing efficiency (η). Hydrogels are an excellent model system in which to study spatial variation in immunoprobing because hydrogels can be fabricated with controlled porosities, measurable partition coefficients^9^, and specific concentrations of immobilized target. We demonstrate that sandwiching a hydrated gel against a thin layer of probe solution (a commonly-used method of probe introduction^5,19,20^) distributes antibody nonuniformly across the chip. We apply bimolecular binding theory to identify a regime within standard IHC and in-gel immunoassay conditions in which η is highly sensitive to local antibody probe concentration, even when the antibody is in excess compared to the antigen. For experimental validation, we develop a stirring strategy which homogenizes antibody probe concentration across the area of the chip without requiring any increase in antibody concentration or volume. This stirring strategy allows us to test controlled boundary conditions while maintaining the same assay format, to compare intra-assay spatial variation in η in chips probed with uniform and nonuniform antibody fluid layers. Using polyacrylamide gels with photopatterned protein spots as well as single-cell immunoblots^7^, we demonstrate significant intra-assay spatial variation in η and background fluorescence when antibody probe is distributed nonuniformly across the assay, despite the antibody being in excess. We establish for the first time, to our knowledge, that probe is nonuniformly distributed across large-format chips immediately after probe is interfaced with the chip (before any spatial variation in partitioning, depletion, or other factors could have an effect). Using both a bimolecular binding model and a controlled hydrogel system, we identify the regime in which this nonuniformity impacts η.

## Methods

### Chemicals/Reagents

Acrylamide/bis-acrylamide 30% solution (37.5:1, A3699), sodium deoxycholate (D6750), sodium dodecyl sulfate (SDS; L3771), and Triton X-100 (X100) for cell lysis buffer, ammonium persulfate (A3678) and N,N,N′,N′-Tetramethylethylenediamine (T9281) for gel polymerization, dichlorodimethylsilane (440272) and 3-(trimethoxysilyl)propyl methacrylate (440159) for wafer and glass silanization, respectively, and bovine serum albumin (BSA, A7030) were all purchased from Sigma-Aldrich. N-(3-((3-benzoylphenyl)formamido)propyl) methacrylamide (BPMAC) was custom-synthesized by PharmAgra Labs. Gels were cast on wafers (WaferPro C04009) microfabricated with SU-8 3050 photoresist (Kayaku Advanced Materials Y311075), coated with dichlorodimethylsilane and gel slick solution (Lonza 50640). 1.5 M Tris-HCl, pH 8.8 (T1588) was purchased from Teknova, 10x tris-glycine buffer (1610734) was purchased from Biorad, and 10x Tris buffered saline with Tween 20 (TBST, 9997 S) was purchased from Cell Signaling Technologies. Purified Turbo GFP (tGFP) protein (FP552) was purchased from Evrogen. Rabbit anti-TurboGFP primary antibody (PA5-22688, lots UC2733591 and UD2749791) and donkey anti-rabbit Alexa Fluor 647 secondary antibody (A31573, lot 1964354) were purchased from ThermoFisher.

### Cell culture

U251 glioblastoma cells were lentivirally infected (multiplicity of 10) to express tGFP. These cells were transfected by and generously provided by Dr. Ching-Wei Chang in Prof. S. Kumar’s Laboratory at UC Berkeley. U251-tGFP cells were cultured in a humidified 37 °C incubator kept at 5% CO2 with DMEM + Glutamax - I medium (ThermoFisher 10566-016) supplemented with 10% fetal bovine serum (Gemini Bio-Products 100–106), 1x non-essential amino acids (ThermoFisher 11140-050), 1 mM sodium pyruvate (ThermoFisher 11360-070), and 100 U/ml penicillin/streptomycin (ThermoFisher 15140-122). Cells were detached with 0.05% Trypsin-EDTA (ThermoFisher 25300-120) and resuspended in 4 °C 1x phosphate-buffered saline to generate cell suspensions used for single-cell immunoblots.

### Single-cell immunoblotting

Single-cell immunoblotting was performed as previously described^20^, with the following modifications. 8%T polyacrylamide gels were chemically polymerized using APS and TEMED on an SU-8 3050 microfabricated mold with microposts (32 μm diameter, ~40 μm height; 800 μm spacing along electrophoretic separation axis, 600 μm spacing between separation lanes). 300 µL of a U251-tGFP cell suspension was pipetted onto a polyacrylamide gel microwell array cast on half a microscope slide (~25 mm ×37.5 mm) and passively settled into microwells.

After cell settling, excess cells were rinsed off the gel, the device was adhered with Vaseline inside a custom-built electrophoresis chamber, and cell lysis (30 s at 4 °C), electrophoresis (20 s at 40 V/cm), and photo-immobilization (300 s at ~20 mW/cm^2^) were performed. Lysis/electrophoresis buffer (1x RIPA: 0.5% SDS, 0.25% sodium deoxycholate, 0.1% Triton X-100, 0.5x Tris-glycine, as previously reported^20^) at 4 °C was used, as this was found to maximize the number of detectable photo-immobilized tGFP bands. We hypothesize that keeping the proteins at 4 °C (rather than the previously-reported 50 °C^20^) minimizes tGFP denaturation and diffusive losses. Immediately after electrophoresis, the device was removed from the electrophoresis chamber and placed gel side up in a 4-well dish with 4 °C 1x TBST and photo-immobilized (OAI Model 30 Collimated Ultraviolet [UV] Light Source). Proteins were photo-immobilized using a collimated UV source to ensure that UV intensity, and thus tGFP photobleaching rate, is spatially-uniform. It is important to wipe Vaseline off the back of the slide prior to photo-immobilization and ensure no bubbles are trapped between the UV source and the gel, as these will make the UV illumination nonuniform by inducing scattering and/or lensing artifacts. After photo-immobilization, the gels were washed for ≥30 minutes in 1x TBST on a rotator, rinsed with dI water, dried with a nitrogen stream, and imaged with the 488 nm laser channel of a fluorescence microarray scanner (Genepix 4300 A, Molecular Devices) to image photo-immobilized tGFP bands.

After collecting images of the photo-immobilized tGFP, gels were rehydrated in 1x TBST and immunoprobed for tGFP. The immunoprobing sequence consisted of primary antibody probe incubation (2 h), wash (2 × 30 min in 1x TBST), secondary antibody probe incubation (1 h), wash (2 × 30 min in 1x TBST). Afterward, gels were rinsed with dI water, dried, and imaged again using the 635 nm laser channel to detect immunoprobed signal.

### Antibody probe introduction methods and imaging

A 40 µL droplet of antibody probe solution was pipetted onto a clean glass plate and a half slide gel (hydrated in 1x TBST) was placed gel side down on top of the droplet, spreading the droplet across the area of the gel. In some experiments, the antibody fluid layer was stirred by laterally shifting the gel across the antibody solution a distance of ~3 cm in multiple directions 3–4 times. When immunoprobing with an antibody bath, 5 half slides were placed in a slide mailer (Globe Scientific, 513062) with enough antibody solution to cover the top of the slides (10 mL). 0.05 mg/ml primary and secondary antibody solutions (diluted in 1x TBST with 2% wt/vol BSA) were used in all experiments.

To image antibody probe distribution across a half slide, fluorescently-labeled secondary antibody incubations were set up against a 50 mm × 75 mm glass slide using polyacrylamide gels with the same composition and dimensions as were used for single-cell immunoblotting separations, except without microwells. The polyacrylamide gels did not contain any photo-immobilized protein. Widefield fluorescence (Cy5 filter cube, Chroma 49009) images of the gel were taken with an Olympus 4x/0.13 NA objective on an Olympus IX71 inverted epifluorescence microscope, with a Lumen Dynamics X-cite exacte fluorescence illumination source coupled to a liquid light guide (Lumatec, 805-00038). Images were stitched in ImageJ.

### Creating and immunoprobing photopatterned protein spots

0.005 mg/ml purified tGFP protein was diffused into an 8%T polyacrylamide gel using the same ‘sandwich’ introduction method as described above for antibodies. After incubating for 1 h, the gel was briefly dipped in dI water to remove excess tGFP pooled on the surface of the gel, and then placed gel side down against a #1.5 H glass coverslip (Ibidi 10812). The gel was laterally shifted against the coverslip to remove any bubbles which would scatter or lens UV. The coverslip-gel assembly was placed on top of a mylar mask (coverslip side down) and exposed to collimated UV light for 300 s at ~20 mW/cm^2^. tGFP is only photo-immobilized in regions of the gel exposed to UV (i.e., regions of the gel which are over clear parts of the mask). Thus, the size, spacing, and number of protein spots is highly tunable, as has been shown with other hydrogel photopatterning methods^21,22^. After photo-immobilization, gels were washed in 1x TBST for 2 × 30 min to remove non-immobilized protein from the gel.

We can estimate the concentration of target antigen and antibody probe within the gel based on the partition coefficients of antigen and antibody into the gel, as well as the antigen photo-immobilization efficiency (see Supplementary Notes S1 and S2). When immunoprobing photopatterned protein spots, we utilize a 23.5:1 ratio of antibody probe:target antigen to ensure antibody is in excess.

### Image and statistical analysis

Fiji (ImageJ version 1.52p, https://imagej.net/Fiji)^23,24^ was used to generate all fluorescence micrographs (i.e., separation lanes and photopatterned protein images); the Grid/Collection stitching plugin^25^ in Fiji was also used to stitch widefield fluorescence microscopy images. All other image analysis and plot generation were performed with MATLAB R2018b (https://www.mathworks.com/products/matlab.html). Area under the curve (AUC) fluorescence of photo-immobilized tGFP bands and immunoprobed protein bands was calculated using custom MATLAB scripts, as previously described^20^. Briefly, regions of interest were defined around each protein band, and a Gaussian function was fit to the background-subtracted intensity profile of each region of interest. AUC values were calculated by summing the intensity profile values within the peak center ±2 standard deviations. As quality control, AUCs of intensity profiles with a signal-to-noise ratio <3 or a Gaussian fit r^2^** <** 0.7 were disregarded. Only separation lanes with AUC values which passed quality control standards for both measurements (photo-immobilized AUC and immunoprobed AUC) were considered for calculation of η and Bland-Altman analysis. Heatmaps were generated using the *imagesc* function in MATLAB, and beeswarm plots were generated using the *plotspread* function^26^.

Statistical analysis was performed using the nonparametric test functions *kruskalwallis* (Kruskal-Wallis test), *multcompare* (post-hoc Tukey test), and *ranksum* (2-tailed Mann-Whitney U test) in MATLAB R2018b. Nonparametric tests were chosen because the sample sizes of data being compared were too small to make distribution assumptions. All error values reported following a ± sign are standard deviations (not standard errors of the mean). Results were determined to be statistically significant if the statistical test yielded a p-value of less than 0.05.

Microsoft Powerpoint version 16.0 (https://products.office.com/en-us/powerpoint) was used to generate all schematics and compile figures.

## Results and Discussion

### η depends on local antibody probe concentration and affinity

**Figure 1:**
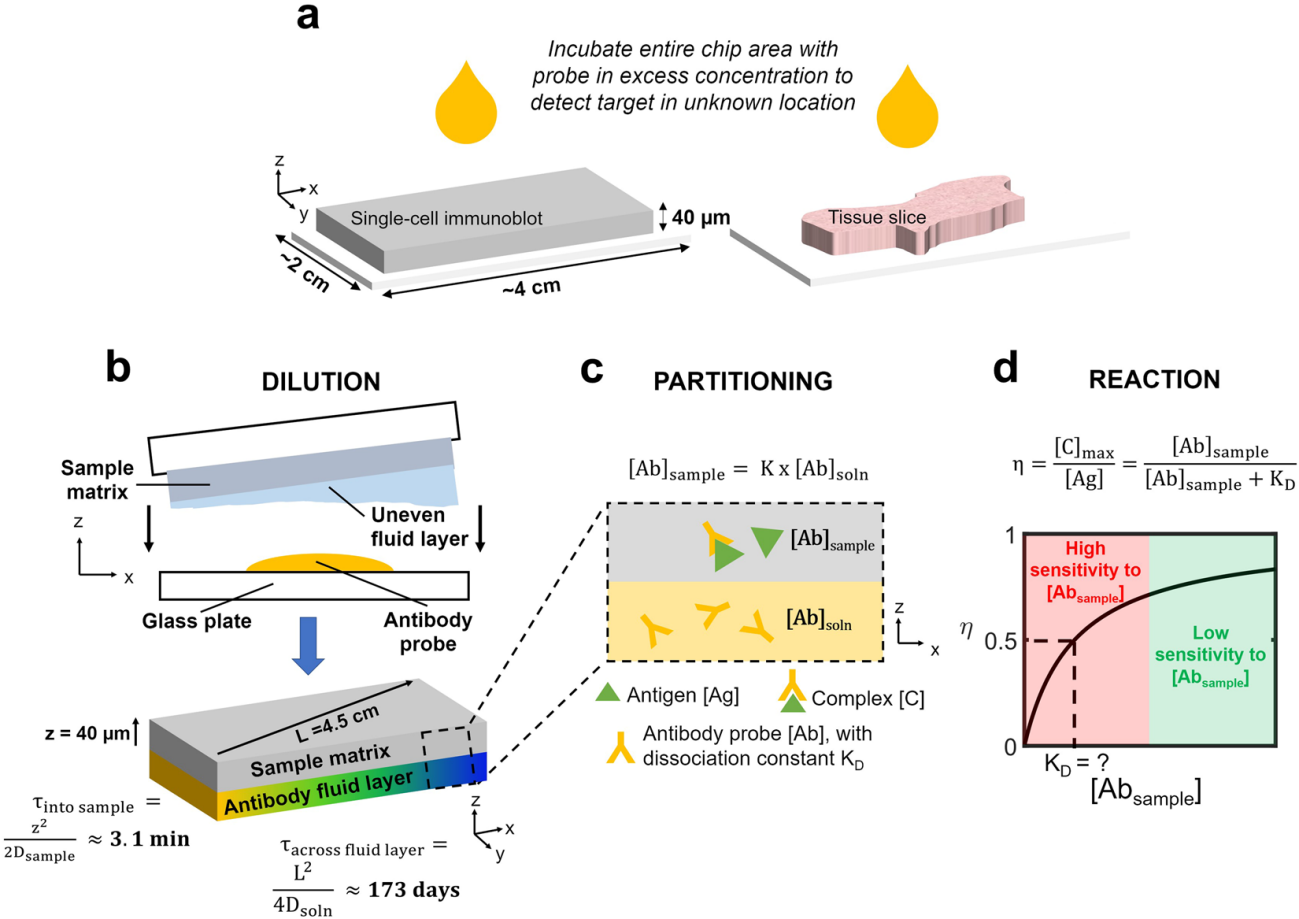
Critical parameters influencing and affected by local antibody probe concentration in large-format chips. (**a**) Two examples of large-format chips: single-cell immunoblot and immunohistochemistry. In both systems, target molecules are immobilized in unknown locations withing a sample matrix (10s of µm thick, centimeters long) and must be incubated with concentrated probe solution for detection. (**b**–**d**) Physicochemical phenomena which influence immunoprobing efficiency in these assays. (**b**) Method of distributing a thin antibody fluid layer across a hydrated sample surface may nonuniformly dilute the antibody. Lateral spatial variation in antibody concentration will not equilibrate over assay immunoprobing timescales because the diffusive timescale of antibody across the lateral length scale (L) of the assay (τ_across fluid layer_) is much greater than the diffusive timescale of antibody into the sample matrix (τ_into sample_). Here, τ_into sample_ is calculated using the diffusivity of antibody in an 8%T polyacrylamide gel. (**c**) Equilibrium antibody concentration in a porous sample ([Ab]_sample_) is governed by the partition coefficient (K) of antibody into the sample and the antibody concentration at the free solution-sample boundary ([Ab]_soln_). (**d**) η is strongly dependent on the concentration of antibody in the sample when the concentration is near the antibody dissociation constant (K_D_), even when antibody is in excess compared to antigen.

We sought to apply bimolecular binding theory to understand the sensitivity of η to two key immunoprobing parameters: antibody probe concentration and affinity. We consider an example system in which a chip with ~cm lateral dimensions and ~µm thickness is sandwiched against a thin layer of antibody solution (see Fig. 1a,b). These lateral and/or axial dimensions are characteristic of DNA microarrays^1^, reverse-phase protein microarrays^3,27^, single-cell immunoblots^7^, IHC/ISH/imaging mass cytometry staining^28^, and other high-throughput assays^29^. We focus our study on IHC, in-gel immunoassays, and other large-format chips in which antibody probes (in excess concentration) bind to targets (in limited concentration) immobilized within a hydrogel, tissue slice, or other 3D matrix which we call the ‘sample’. In these systems, three primary phenomena influence antibody probe binding: dilution, partitioning, and reaction. First of all, samples are typically incubated with a small (10s of µL) volume of antibody to conserve reagents; as a result, however, the fluid layer on the hydrated samples may non-negligibly dilute the antibody (see Fig. 1b)^12^. The concentration of antibody reaching targets within the sample is further limited by partitioning, the phenomenon in which solute concentration in a material may be lower than in free solution due to size-exclusion from pores or other factors (see Fig. 1c)^30^. Ultimately, η depends on antibody-antigen reaction (see Fig. 1d). To study the interplay of these factors, we chose hydrogels as a model system because we can precisely pattern target antigen within the gel, as well as control hydrogel density, which governs antibody probe partitioning into the gel. Hydrogels are also a valuable biosensing platform, as hydrogels offer unique advantages as compared to planar substrates, such as minimal fouling, facile functionalization, and higher-capacity molecular capture within a 3D volume^31–34^.

Immunoprobing time is governed by the sum of the characteristic timescales of antibody transport into the sample (${{\rm{\tau }}}_{{\rm{into}}{\rm{sample}}}$) and antibody binding (${{\rm{\tau }}}_{{\rm{rxn}}}$). Antibody transport into the sample is dependent on the thickness of the sample (z) and diffusivity of the antibody in the sample (D_sample_), according to Eq. (1)**:**

1 $${{\rm{\tau }}}_{{\rm{into}}{\rm{sample}}}=\frac{{{\rm{z}}}^{2}}{2{{\rm{D}}}_{{\rm{sample}}}}$$

In the case of a model 40 µm thick, 8%T polyacrylamide gel with D_gel_ of 4.3 × 10^−12^ m^2^ s^−1^ as previously reported^7^, ${{\rm{\tau }}}_{{\rm{into}}{\rm{gel}}}$ ≈ 3.1 min. Antibody reaction time is dependent on the kinetics of the antibody (k_on_ and k_off_ rates) as well as the local antibody concentration in the sample, according to Eq. (2)**:**

2 $${{\rm{\tau }}}_{{\rm{rxn}}}=\frac{1}{{{\rm{k}}}_{{\rm{on}}}{[{\rm{Ab}}]}_{{\rm{sample}}}+{{\rm{k}}}_{{\rm{off}}}}$$

For an intermediate-affinity antibody (k_on_ = 10^5^ M^−1^ s^−1^, k_off_ = 10^−4^ s^−1^) and [Ab]_gel_ of 10 nM, ${{\rm{\tau }}}_{{\rm{rxn}}}$ ≈ 15 min. Thus, taking into consideration both antibody transport and reaction times (4 τ each), immunoprobing protein captured in a gel tens of µm thick takes ~1.2 h.

To understand whether antibodies in the fluid layer would equilibrate laterally over standard immunoprobing timescales, we also estimated the timescale of antibody transport across the fluid layer. This timescale is dependent on the diffusivity of the antibody in free solution (D_soln_) and the lateral length scale of the fluid layer (L), according to Eq. (3)**:**

3 $${{\rm{\tau }}}_{{\rm{across}}{\rm{fluid}}{\rm{layer}}}=\frac{{{\rm{L}}}^{2}}{4{{\rm{D}}}_{{\rm{soln}}}}$$

For L = 45 mm and D_soln_ of 3.4 × 10^−11^ m^2^ s^−1^ as previously reported^35^, ${{\rm{\tau }}}_{{\rm{across}}{\rm{fluid}}{\rm{layer}}}$ = 173 days, suggesting that the lateral concentration profile of antibody in the fluid layer (and thus, in the gel) will not reach equilibrium during immunoprobing. Increasing the temperature of the fluid layer to increase antibody diffusivity does not sufficiently reduce the diffusive timescale; for example, at 37 °C, ${{\rm{\tau }}}_{{\rm{across}}{\rm{fluid}}{\rm{layer}}}$ is still 98 days (see Supplementary Note S3). Thus, any initial lateral spatial variation in antibody concentration across large-format chips such as IHC and single-cell immunoblots will not equilibrate, introducing spatial variation in η in certain regimes of antibody affinity and concentration. η depends on [Ab]_sample_ and the antibody dissociation constant (K_D_) according to Eq. (4) ^16^**:**

4 $${\rm{\eta }}=\frac{{[{\rm{C}}]}_{{\rm{\max }}}}{[{\rm{Ag}}]}=\frac{{[{\rm{Ab}}]}_{{\rm{sample}}}}{{[{\rm{Ab}}]}_{{\rm{sample}}}+{{\rm{K}}}_{D}}$$

where [C]_max_ is the maximum concentration of immunocomplex formed and [Ag] is the antigen concentration in the sample. From this relationship, it is evident that η is highly sensitive to variation in [Ab]_sample_ when K_D_ ≈ [Ab]_sample_, even when the antibody is in excess compared to antigen (see Fig. 1d).

[Ab]_sample_ in IHC and in-gel immunoassays falls within the same range as reported antibody K_D_ values, making it likely that assays operate in the regime where η is highly sensitive to variation in [Ab]_sample._ In IHC, tissue slices are typically incubated with antibody probe concentrations of ~10s of nM^36,37^. In single-cell immunoblotting, hydrogels are incubated with ~67–333 nM antibody probe^20^, but in-gel antibody concentrations are much lower (~10 nM) due to thermodynamic partitioning^9^ (see Supplementary Note S2). K_D_ of commercial antibodies span many orders of magnitude, from fM to µM^14,15,18^, thus encompassing typical [Ab]_sample_ levels. However, it is typically impossible to determine whether an assay is operating in the K_D_ ≈ [Ab]_sample_ regime without substantial additional characterization. K_D_ of commercial antibodies is not typically reported and can vary from lot to lot^17,38^. While K_D_ can be measured using techniques such as surface plasmon resonance, enzyme linked immunosorbent assays, and kinetic polyacrylamide gel electrophoresis^39^, K_D_ values measured by these techniques can vary by orders of magnitude due to run-to-run variability and differences in measurement conditions which may not match the system of interest (e.g., binding in solution vs. on a surface)^39,40^. [Ab]_sample_ may also be unknown, as antibody penetration into tissue sections for IHC has been found to be nonuniform and variable^10^. Increasing probe concentration to try to avoid the K_D_ ≈ [Ab]_sample_ regime is often cost-prohibitive, as thermodynamic partitioning limits the proportion of antibody which will diffuse into nanoporous samples such as hydrogels. For example, to ensure [Ab]_sample_ > K_D_ (and thus, η is relatively insensitive [Ab]_sample_) in the case where K_D_ = 1 µM, we estimate that one single-cell immunoblot would need to be incubated with >2 mg each of primary and secondary antibody (>US$9,000 total, at the time of publication) (see Supplementary Note S4). Thus, because it is challenging to avoid a regime in which η is sensitive to spatial variation in [Ab]_sample_, we instead sought to investigate strategies to minimize spatial variation in [Ab]_sample_.

### Characterizing and controlling antibody probe distribution at the sample-fluid layer interface

**Figure 2:**
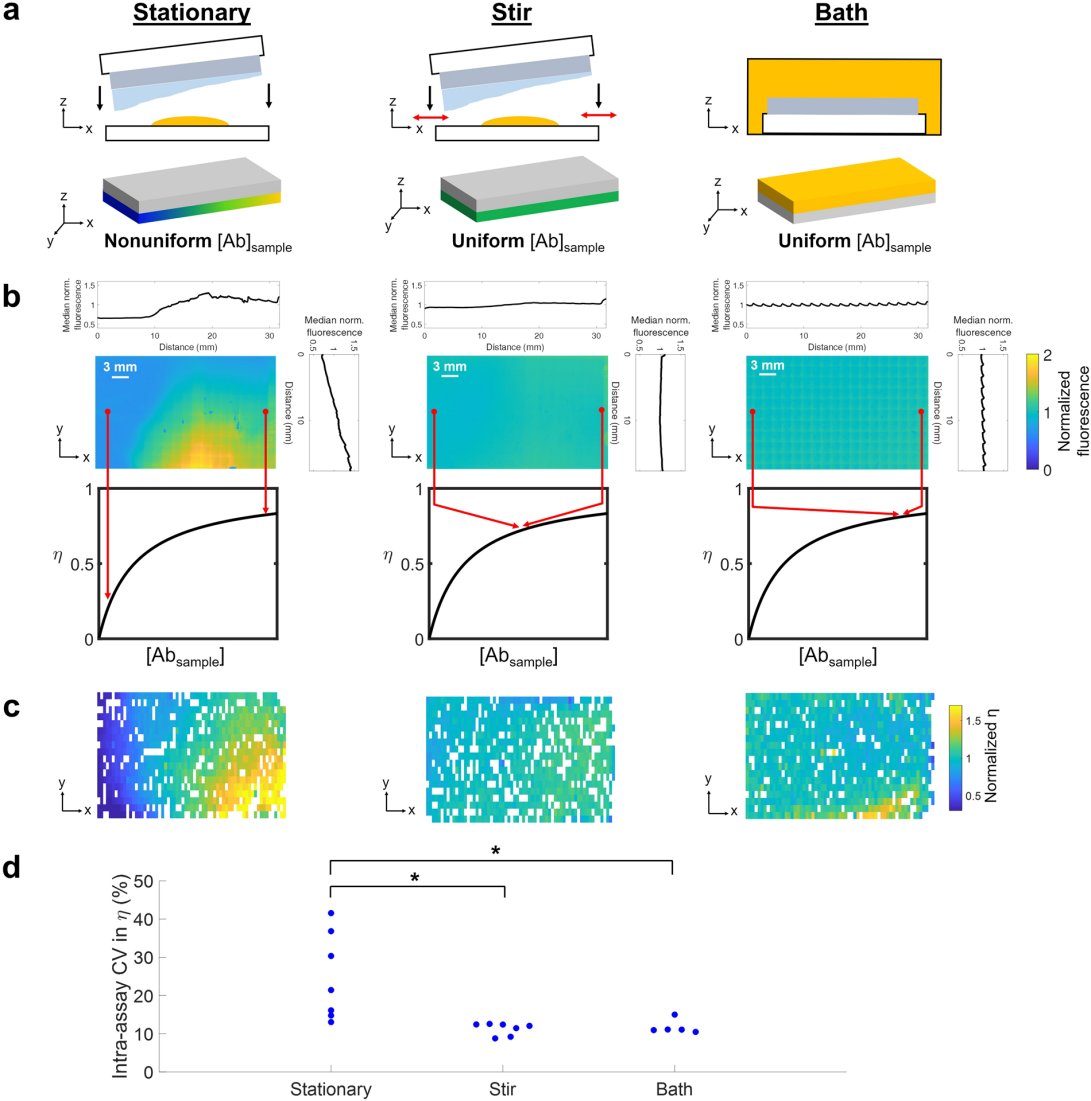
Intra-assay spatial variation in antibody probe distribution and η in three immunoprobing configurations yielding different concentration boundary conditions: a stationary antibody fluid layer, a stirred antibody fluid layer, or an antibody bath. (**a**) We hypothesize that a stationary antibody fluid layer will have lateral spatial variation in antibody concentration due to nonuniform dilution by an uneven fluid layer on the gel. We also hypothesize that stirring the antibody fluid layer by shifting the gel laterally will homogenize the fluid layer to a similar extent as an antibody bath (positive control). (**b**) Representative heatmaps of antibody fluorescence across the fluid layer, normalized to the mean fluorescence intensity within each image. Median intensity profiles in the x- and y- directions demonstrate that spatial nonuniformity in antibody concentration is greatest in the stationary antibody fluid layer. Bimolecular binding modeling shows that if K_D_ ≈ [Ab_sample_], spatial variation in antibody distribution yields variation in η. (**c**) Representative heatmaps of η of photopatterned tGFP spots immunoprobed with a stationary antibody fluid layer, stirred antibody fluid layer, or antibody bath (chips in (**c**) were *not* probed with the antibody fluid layers shown in (**b**); the spatial patterns are not directly comparable). Each rectangle in the heatmap represents one tGFP spot; white rectangles are spots which did not pass quality control standards and thus do not have quantifiable η. (**d**) Beeswarm plot of intra-assay CV in η (Kruskal-Wallis test, p = 0.0033; post-hoc Tukey test, p_stationary vs. stir_ = 0.0091, p_stationary vs. bath_ = 0.0131).

We hypothesize that spatial variation in local antibody probe concentration can arise when sandwiching a hydrated large-format chip with a droplet of antibody solution, via nonuniform local dilution of antibody due to an uneven fluid layer on the hydrated gel, tissue slice, or other sample containing immobilized target (see Fig. 2a). Because these samples are only ~10s of µm thick and are often incubated with small volumes (~10s of µL) of probe solution^5,12,20^, any fluid layer on the sample can substantially impact local antibody probe concentration. For example, excess buffer remaining on an IHC sample prior to primary antibody incubation has been observed to add variation to antibody dilution, although the effect on lateral uniformity was not characterized^12^. Nonuniform local antibody dilution would lead to differences in antibody concentration boundary conditions (at the sample-fluid layer boundary) in different regions of the immunoassay, which can result in intra-assay technical variation in η.

To determine whether local antibody concentration varies across a large-format chip, we characterized intra-assay variation in antibody concentration in a model system in which a hydrated ~22 × 35 mm polyacrylamide gel was sandwiched against a 40 µL droplet of fluorescently-labeled antibody solution (i.e., ‘stationary’ configuration). To do so, we used widefield fluorescence microscopy to image the antibody fluid layer sandwiched against the gel. Median fluorescence intensity varied by 60% ± 24% along the x axis and 77% ± 13% along the y axis (n = 3 gels). To estimate the degree to which antibody concentration would differ between individual target spots on a large-format chip, we also divided the antibody fluorescence micrograph into 500 µm x 1000 µm ‘analysis regions’. In the stationary configuration, mean fluorescence of individual analysis regions within a single chip differed by up to 2.88-fold (±0.13-fold). Thus, we observed substantial variation in antibody fluorescence (a proxy for antibody concentration) across the gel (see Fig. 2b).

To determine the impact of the observed nonuniform antibody probe distribution, we sought to develop a method to control the boundary condition at the gel-antibody solution interface. By controlling the boundary condition, we could compare intra-assay variation in η in configurations where antibody was either uniformly or nonuniformly distributed. Numerous microscale fluid mixers based on heating^41^, oscillation^42^, pneumatics^5^ and electrokinetics^43^ have been developed and applied to probe solutions in microarray^5^, immunoassay^43^, and automated immunohistochemistry platforms^44^ to speed up reactions and increase staining uniformity. However, the improvement in uniformity has not been substantially characterized. Additionally, we sought a method to control the initial boundary condition that minimally altered assay format, to facilitate comparison of nonuniform and uniform antibody concentration boundary conditions. With these considerations in mind, we hypothesize that stirring the antibody fluid layer by laterally shifting the gel over the antibody fluid layer will homogenize the concentration of the antibody fluid layer (i.e., ‘stirred’ configuration). By imaging the antibody fluid layer after each successive stirring movement, we find that the antibody fluid layer becomes well-mixed after moving the gel ~2–3 cm once in each of 4 different directions (see Supplementary Fig. S1). Indeed, in the stirred configuration, intra-assay fluorescence varied by 35% ± 10% in the x axis and 15 ± 4.1% in the y axis (n = 3 gels), which is substantially lower variation than in the stationary configuration (see Fig. 2b). Likewise, mean fluorescence (i.e., antibody concentration) of analysis regions within a stirred gel exhibited smaller region-to-region fold-change differences of up to 1.50 ± 0.19 for the stirred configuration as compared to almost 3-fold in the stationary configuration.

As a positive control for a homogeneous boundary condition, we also characterized antibody distribution across a gel immersed in an antibody bath. We expect that bath immersion will yield a uniform antibody concentration boundary condition, as any fluid layer (volume of µLs) on the sample is now negligible as compared to the antibody bath solution (volume of mL) and will not induce substantial antibody dilution. Additionally, the thickness of the antibody fluid layer bordering the gel is on the order of mm in the bath, rather than on the order of µm in the stationary or stirred configurations, which facilitates concentration equilibration. In an antibody bath, lateral variation in antibody concentration at the gel boundary is quickly homogenized by diffusion of antibodies from the thicker antibody fluid layer (length scale ~ µm, diffusive τ ~ min) to the gel boundary, rather than by lateral diffusion of antibodies across the fluid layer (length scale ~ mm, diffusive τ ~ days). As expected, we found that antibody concentration variation across the hydrogel was low in the bath configuration (see Fig. 2b); median fluorescence varied by 13% ± 2.0% in the x axis, 8.8% ± 1.5% in the y axis (n = 3 gels). Mean fluorescence of analysis regions within the bath configuration differed by up to 1.20-fold (±0.04-fold), the smallest difference among the three immunoprobing configurations characterized. However, the bath configuration requires a ~50x larger volume of antibody probe solution than the stationary and stirred configurations. Due to the high antibody concentrations typically required due to thermodynamic partitioning of antibody into nanoporous hydrogels, routine bath immunoprobing would be extremely costly. For example, in the model system described here, the stationary or stirred configurations require ~ $8 of primary anti-tGFP antibody per gel while the bath configuration requires ~$400 of the same antibody. Overall, we have observed substantial spatial variation in antibody probe concentration when a large-format hydrogel is sandwiched against antibody solution, a phenomenon we hypothesize is due to uneven antibody dilution by a nonuniform fluid layer on the hydrogel. We have also demonstrated methods to control the concentration boundary condition to investigate the relationship between intra-assay variation in antibody concentration and η in a precise manner.

### Spatial variation in antibody probe concentration yields variation in η even when antibody is in excess

After observing intra-assay variation in antibody concentration across a stationary antibody fluid layer, we next sought to test our hypothesis that intra-assay variation in η is greater in the stationary configuration (with nonuniform boundary condition) than in the stirred configuration (with a more uniform boundary condition). Variable antibody concentration at the boundary will cause intra-assay variation in η if K_D_ ≈ [Ab_sample_], yielding intra-assay technical variation in protein abundance measurements. To investigate whether intra-assay variation in η is higher in the stationary configuration, we photopatterned ~1100 tGFP spots on a ~22 mm × 35 mm gel and immunoprobed using an excess of antibody probe (see Supplementary Notes S1 and S2) and measured η based on the ratio of the AUCs of immunoprobed and photo-immobilized spots (see Supplementary Fig. S2). Because tGFP is fluorescent, photo-immobilized tGFP spots (prior to immunoprobing) can be detected via fluorescence imaging. The relative photo-immobilized and immunoprobed tGFP signal is quantified as the AUC of the fluorescence intensity profile. Experimentally, η is defined as Eq. (5):

5 $${\rm{\eta }}=\frac{{\rm{Immunoprobed}}\,{\rm{AUC}}}{{\rm{Photo}}\mbox{--}{\rm{immobilized}}\,{\rm{AUC}}}$$

We observed the largest intra-assay spatial variation in η in the gels immunoprobed with a stationary antibody fluid layer (see Fig. 2c). To quantify the level of intra-assay variation, we calculated the coefficient of variation (CV) in η within each gel and found that the CV was significantly higher in the stationary immunoprobing configuration (n = 7 gels) as compared to the stirred (n = 7 gels) and bath (n = 5 gels) immunoprobing configurations (Kruskal-Wallis test, p = 0.0033; post-hoc Tukey test, p_stationary vs. stir_ = 0.0091, p_stationary vs. bath_ = 0.0131), supporting our hypothesis (see Fig. 2d).

### Bland-Altman analysis quantifies degree of agreement between replicates

**Figure 3:**
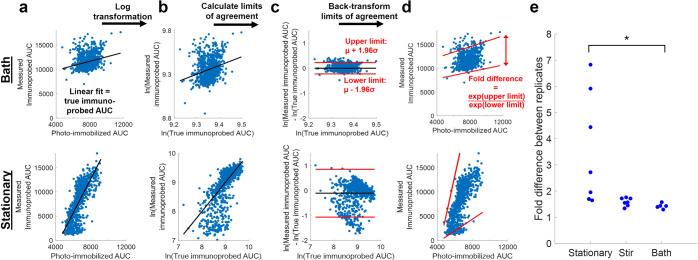
Quantifying the degree of agreement between photo-immobilized and immunoprobed AUC of photopatterned tGFP spots as a measure of intra-assay technical variation. Bland-Altman analysis is used to obtain a measure of technical variation in the stationary, stirred, and bath immunoprobing conditions. (**a**) Photo-immobilized AUC values are mapped to the immunoprobed AUC scale using the linear fit of photo-immobilized and immunoprobed data. (**b**) True immunoprobed AUCs (based on the linear fit mapping of photo-immobilized AUC) and measured immunoprobed AUCs are log-transformed to facilitate subsequent data analysis and interpretation. The line of equality (y = x) is also shown to indicate what the data would look like in the absence of technical variation. (**c**) The differences between the log-transformed measured and true immunoprobed AUCs are plotted against protein abundance. The limits of agreement are calculated based on the mean (µ) and standard deviation (σ) of these differences. (**d**) Data and limits of agreement are back-transformed by taking the anti-log of each value. (**e**) Fold difference in measured immunoprobed signal which arises from two replicate photo-immobilized protein spots, based on Bland-Altman analysis. Each point on the beeswarm plot is a replicate assay; each assay contains ~1100 photopatterned tGFP bands. Gels immunoprobed with a stationary antibody fluid layer have significantly greater technical variation as compared to the bath immunoprobing configurations (Kruskal-Wallis test, p = 0.0039; post-hoc Tukey test, p_stationary vs. bath_ = 0.0032).

We sought to quantify intra-assay technical variation in terms of the expected measurement error of immunoprobed AUC. While CV of η provides a measure of overall intra-assay variation, it does not quantify the expected measurement error of any individual spot. We apply Bland-Altman analysis to quantify the degree of agreement between photo-immobilized and immunoprobed measurements. Bland-Altman analysis defines limits of agreement between two paired datasets based on the distribution of differences in the datasets^45^. Photo-immobilized AUC is a measure of the true amount of protein in the gel, while the immunoprobed AUC is convolved with technical variation in η. Because photo-immobilized and immunoprobed AUCs were measured in different fluorescence channels and thus have different scales, we used a linear fit between photo-immobilized and immunoprobed data to map each photo-immobilized AUC to the immunoprobed AUC scale. We consider the immunoprobed AUC predicted by the linear fit from each photo-immobilized data point as the *true* immunoprobed AUC; we consider the AUC measured from immunoprobed signal intensity as the *measured* immunoprobed AUC (see Fig. 3a). True and measured immunoprobed AUCs were then log transformed (see Fig. 3b) and limits of agreement were calculated as described previously (see Fig. 3c)^45^. Due to the logarithm quotient rule, the difference in logarithms (i.e., limits of agreement of log-transformed data) equals the logarithm of a quotient (i.e., the ratio between measured and true immunoprobed AUC). Thus, we can back-transform the limits of agreement to determine the minimum and maximum percentage difference between true and measured immunoprobed AUC (see Fig. 3d). The ratio of the maximum and minimum percentage difference yields the possible measured fold difference between replicates. For example, a fold difference of 3 means that the immunoprobed AUC of two replicate protein spots with identical photo-immobilized AUC would differ by up to 3-fold.

Here, we do not aim to evaluate whether true and measured immunoprobed AUCs are equivalent (as Bland-Altman analysis has traditionally been used for). Instead, we use Bland-Altman analysis to quantify the intra-assay technical variation, in order to compare amount of error introduced with stationary, stir, and bath immunoprobing. Applying Bland-Altman analysis to the photopatterned immunoprobing data in Fig. 2, we find that the measured fold difference between replicates is significantly higher when photopatterned tGFP spots are immunoprobed with a stationary antibody fluid layer (fold difference = 3.60 ± 2.14, n = 7 gels) as compared to an antibody bath (fold difference = 1.42 ± 0.10, n = 5 gels) (Kruskal-Wallis test, p = 0.0039; post-hoc Tukey test, p_stationary vs. bath_ = 0.0032) (see Fig. 3e). This result supports our hypothesis that immunoprobing with a stationary antibody fluid layer increases technical variation in immunoprobing measurements due to greater intra-assay variation in antibody concentration. While the stirred configuration (fold difference = 1.59 ± 0.16, n = 7 gels) did not have significantly lower fold differences than the stationary configuration, stirring lowers and narrows the range of fold differences as compared to the stationary immunoprobing configuration. Given that protein fold changes of ≤ 3 have been implicated in differential chemotherapeutic response^46,47^, reducing the measured fold difference between technical replicates is key to studying important biological variation^48^.

### Application to single-cell immunoblotting

**Figure 4:**
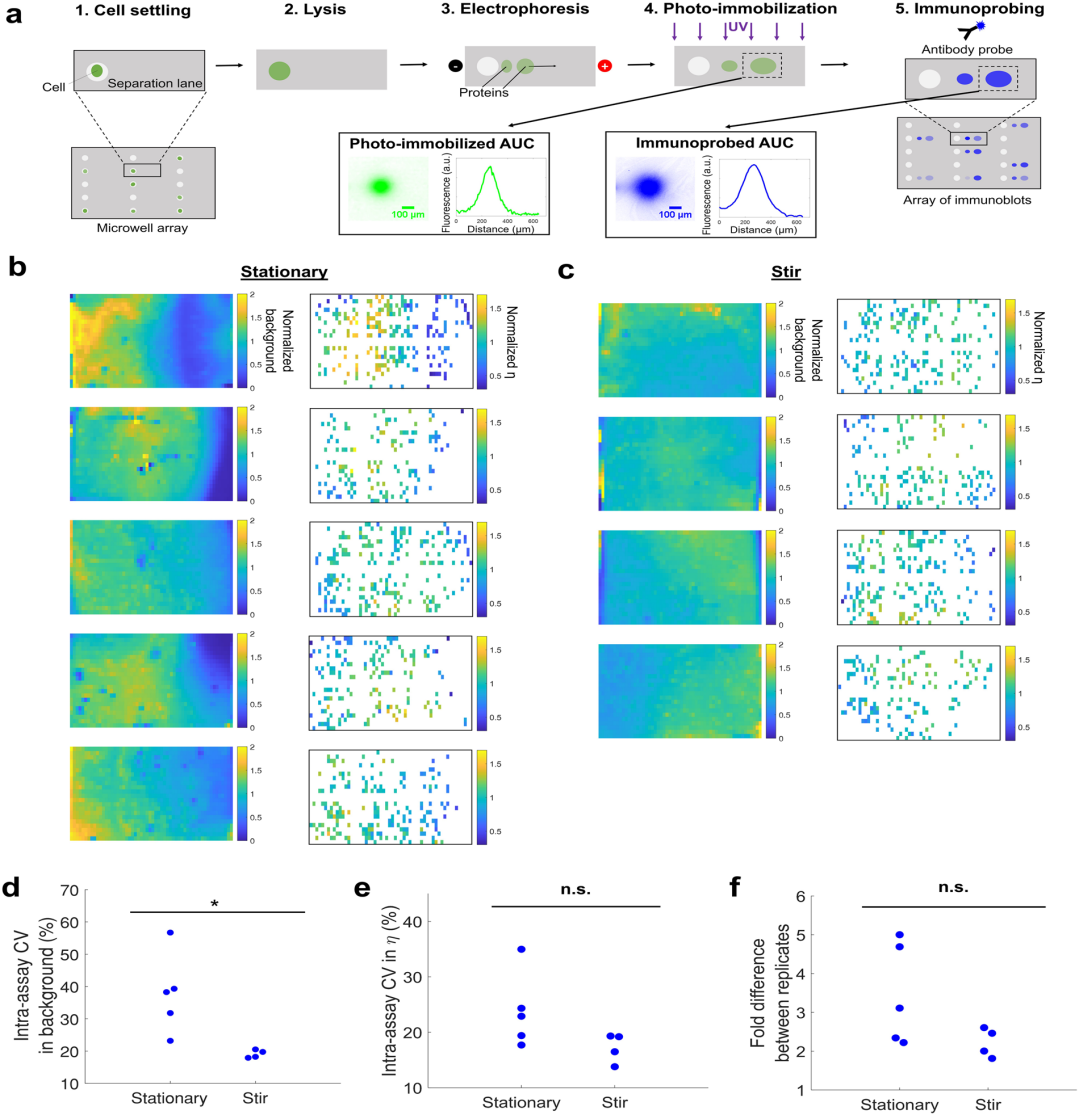
Spatial distribution of background fluorescence and η in a large-format in-gel immunoassay to measure single-cell protein abundance. (**a**) Single-cell immunoblotting workflow. When run with tGFP-expressing cells, upstream measurement of photo-immobilized tGFP abundance can be measured in addition to immunoprobed tGFP abundance, the standard assay readout. (**b**,**c**) Heatmaps of background fluorescence and η in each assay (normalized to the mean within each assay); background was measured in each separation lane, but η is only measured in lanes with a settled cell. Spatial variation in background fluorescence and η is greater when immunoprobing with a (**b**) stationary antibody fluid layer than with a (**c**) stirred antibody fluid layer. (**d**) Intra-assay CV in background fluorescence is significantly higher in assays immunoprobed with a stationary antibody fluid layer than a stirred layer, supporting the hypothesis that in-gel antibody concentration does not diffusively homogenize over immunoprobing timescales (Mann-Whitney U test, p = 0.0159). (**e**) Intra-assay CV in η in assays immunoprobed with a stationary (n = 5) and stirred (n = 4) fluid layer. (**f**) Fold difference in measured immunoprobed signal which arises from two replicate photo-immobilized protein spots, based on Bland-Altman analysis. The fold difference is generally greater for assays immunoprobed with a stationary antibody fluid layer, indicating higher technical variation in η.

We sought to confirm that the dependence of η on local antibody probe concentration observed when immunoprobing photopatterned protein spots also held true in the single-cell immunoblot, an array of in-gel immunoassays across a large-format chip. This large-format chip is designed to measure single-cell protein abundance (see Fig. 4a). Individual U251-tGFP cells were settled into microwells and lysed, and proteins were electrophoresed through the gel and photo-immobilized as previously-reported^20^. Lysis and electrophoresis temperature were tuned to reduce diffusive losses and tGFP denaturation to maximize photo-immobilized tGFP intensity. Gels were subsequently immunoprobed and η was measured for each separation lane containing a cell.

We first investigated whether background intensity of immunoprobed separation lanes was spatially dependent in the different immunoprobing configurations. We hypothesized that the amount of antibody probe retained in the gel after the wash step (due to chemical interactions with hydrogel components^9^ or entropic trapping^49^) would be proportional to the local in-gel antibody concentration, and thus background intensity could provide insight on the antibody concentration distribution across the assay during immunoprobing. Indeed, we observed that background intensity of the immunoprobed separation lanes had a similar spatial pattern as η (see Fig. 4b,c), suggesting that background intensity after probe washout is proportional to local antibody concentration at the gel-fluid boundary during probe incubation (i.e., antibody is proportionally partitioning into the gel and not fully washing out). Intra-assay variation in background intensity is significantly greater in gels probed with a stationary antibody fluid layer (n = 5 gels), as compared to a stirred fluid layer (n = 4 gels) (Mann-Whitney U test, p = 0.0159) (see Fig. 4d). Thus, immunoprobing with a stationary antibody fluid layer yields larger intra-assay variation in antibody concentration both in the fluid layer and in the gel after washout.

The implications of the finding that background intensity is proportional to local antibody concentration differ depending on the immunoprobing regime in which the assay is operating. If K_D_ ≈ [Ab]_sample_, areas with higher local antibody probe concentration will have higher η, but our results suggest these areas will also have higher background after probe washout, creating two opposing effects on the limit of detection. In contrast, if [Ab]_sample_>> K_D_, areas of the sample incubated with higher antibody probe concentration will not have substantially higher η (as in this regime, η is insensitive to [Ab]_sample_). However, areas incubated with higher probe concentrations will retain more antibody after washout, leading to higher background and thus a higher limit of detection. It is important to minimize intra-assay variation in limit of detection so that expression of low-abundance proteins in single cells can be accurately compared. Thus, nonuniform antibody distribution can have multiple detrimental effects on assay performance, by increasing intra-assay technical variation in η and/or increasing the limit of detection, depending on the K_D_ and [Ab]_sample_ regime.

Subsequently, we investigated whether the stationary antibody fluid layer would yield larger intra-assay variation in η in the single-cell immunoblot, as was observed in the photopatterned protein system. While there was no significant difference in intra-assay variation in η between the stationary and stirred immunoprobing configurations, stirring narrows and lowers the range of CVs in η (see Fig. 4e). Similarly, the fold difference in expected immunoprobed AUC from replicate protein spots narrowed and lowered in the stirred system as compared to the stationary system, although not significantly (see Fig. 4f). We hypothesize that the smaller sample size (~100 cells/assay, due to stochastic settling, rather than ~1100 photopatterned protein spots/assay as in Fig. 2) may reduce the statistical power of the single-cell immunoblotting system to detect differences in the immunoprobing methods. Additionally, the exact mechanics of the fluid layer on the hydrated gel are not fully understood. It is likely that some replicate assays had a more uniform fluid layer to begin with, resulting in a more homogeneous local antibody concentration; thus, some stationary immunoprobing replicates may match the more uniform antibody concentration boundary conditions of the stirred fluid layers.

## Conclusions

Here, we pose and investigate a physicochemical mechanism of intra-assay spatial variation for immunoassays performed across large-format chips, specifically focusing on configurations where target is immobilized at unknown locations within a sample matrix, such as in single-cell immunoblotting and tissue section-analysis by IHC. Using fluorescence microscopy, we have characterized the uniformity of antibody probe distribution across a model polyacrylamide gel sample, observing substantial intra-assay spatial variation in antibody concentration which is reduced by laterally shifting the gel to stir the antibody fluid layer. Based on bimolecular binding theory, we hypothesized that for antibody probe concentrations near K_D_, η is highly sensitive to local antibody probe concentration, despite the antibody being in excess as compared to the antigen. Both in photopatterned gels and single-cell immunoblot samples, we find that intra-assay variation in η is generally higher when the antibody fluid layer is not stirred (and thus more nonuniform), supporting our hypothesis. We also apply Bland-Altman analysis to rigorously quantify intra-assay technical variation based on the degree of agreement between photo-immobilized and immunoprobed AUC values. Overall, this research demonstrates that uniform intra-assay probe distribution can be critical to reducing technical variation in large-format chips. This is especially true for in-gel immunoassays and IHC, where it can be difficult to know or adjust the regime one is operating in due to limited antibody probe partitioning into the porous sample and the fact that the binding kinetics of commercial antibodies are rarely reported. If a researcher has a choice of antibodies with known K_D_ values, we recommend selecting an antibody with K_D_ at least an order of magnitude lower than the expected local antibody probe concentration at the position of the target antigen, to avoid being in a regime where η is sensitive to variation in probe concentration. Regardless of whether K_D_ is known or not, we recommend ensuring that antibody probe concentration is spatially uniform via stirring or other microscale mixing techniques, as we have demonstrated here that uniform probe distribution reduces intra-assay technical variation in η and background. By reducing intra-assay technical variation, finer biological differences can be distinguished, facilitating discoveries in a variety of spatially-arrayed assays.

## Supplementary information

Supplementary information.

## Data Availability

The datasets generated and/or analyzed in the current study are available from the corresponding author on reasonable request.
