# Supplementary material for: Probe-target hybridization depends on spatial uniformity of initial concentration condition across large-format chips
Source: Sci Rep. 2020 May 29;10:8768. doi: 10.1038/s41598-020-65563-3 (PMC7260366; doi:10.1038/s41598-020-65563-3)
Supplement: Supplementary file 1 — Supplementary information. [file 41598_2020_65563_MOESM1_ESM.docx]

Supplementary Information

**Probe-target hybridization depends on spatial uniformity of initial concentration condition across large-format chips**

Alisha Geldert^1^, Haiyan Huang^2,3^, Amy E. Herr^1,4^

^1^UC Berkeley – UCSF Graduate Program in Bioengineering, Berkeley, United States

^2^Department of Statistics, University of California Berkeley, Berkeley, California, 94720, United States

^3^Center for Computational Biology, University of California Berkeley, Berkeley, California, 94720, United States

^4^Department of Bioengineering, University of California Berkeley, Berkeley, California, 94720, United States


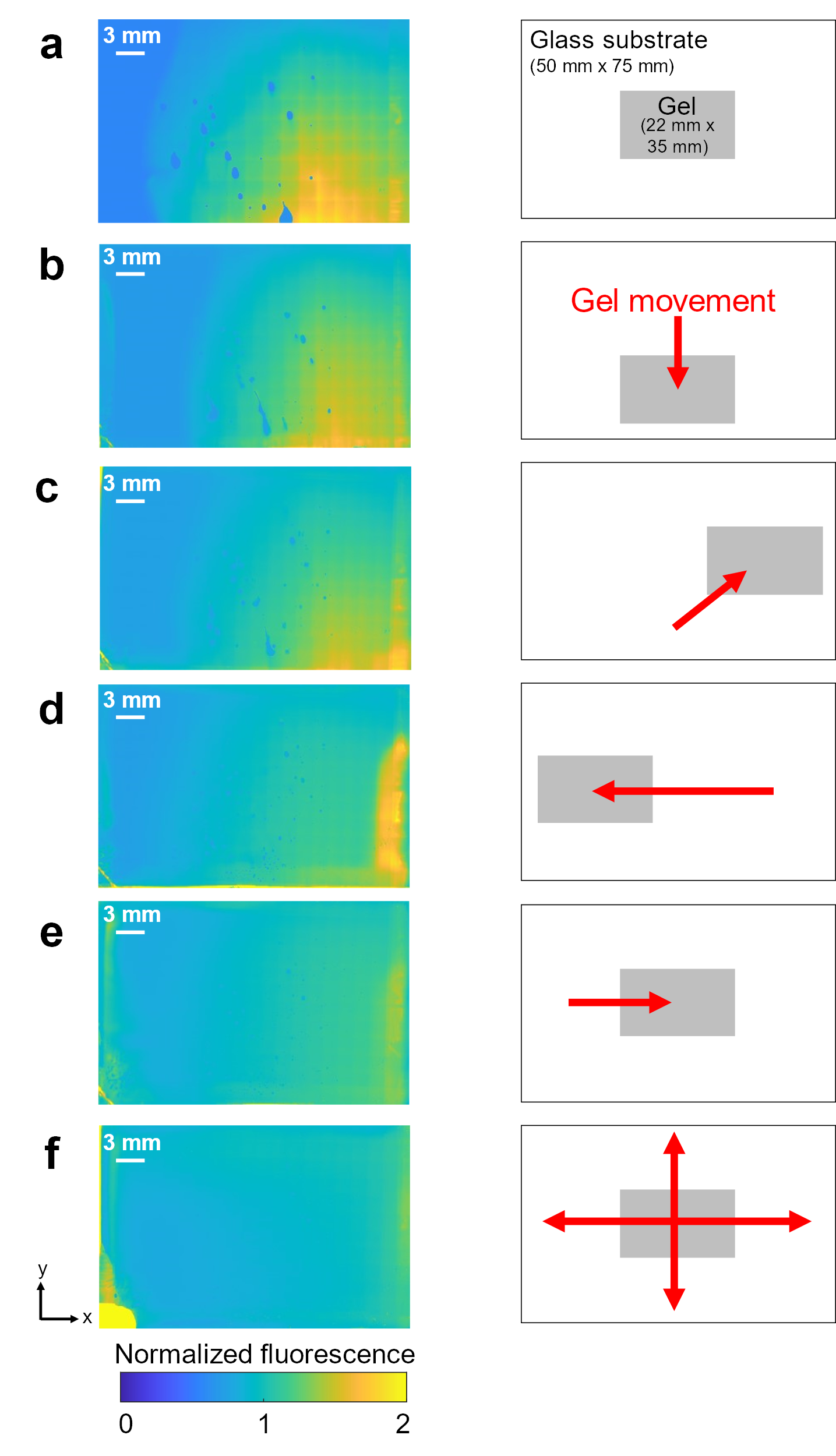


**Figure S1.** Stirring homogenizes antibody probe concentration across centimeter length scales. Widefield fluorescence microscopy images of a fluorescently-labeled secondary antibody fluid layer between a polyacrylamide gel (~22 mm x 35 mm) and glass substrate (50 mm x 75 mm) are displayed as heatmaps of fluorescence intensity (normalized by the mean fluorescence within each image). (a) Intra-assay CV in antibody fluorescence (a proxy for concentration) is 32.8% in the stationary antibody fluid layer (immediately after sandwiching the gel with the antibody). (b-e) Spatial variation in the antibody fluid layer is reduced by stirring the fluid layer by sliding the gel ~2-3 cm laterally across the glass substrate. Heatmaps show antibody distribution after each successive movement. After 4 movements (e), intra-assay CV in antibody fluorescence has dropped to 14.0% and antibody is homogenized to a similar degree as a gel which has been thoroughly stirred (with ~20 movements) (f). Stirring the fluid layer does increase the risk of gel tearing off the glass slide, as is seen in the bottom left corner of (f).


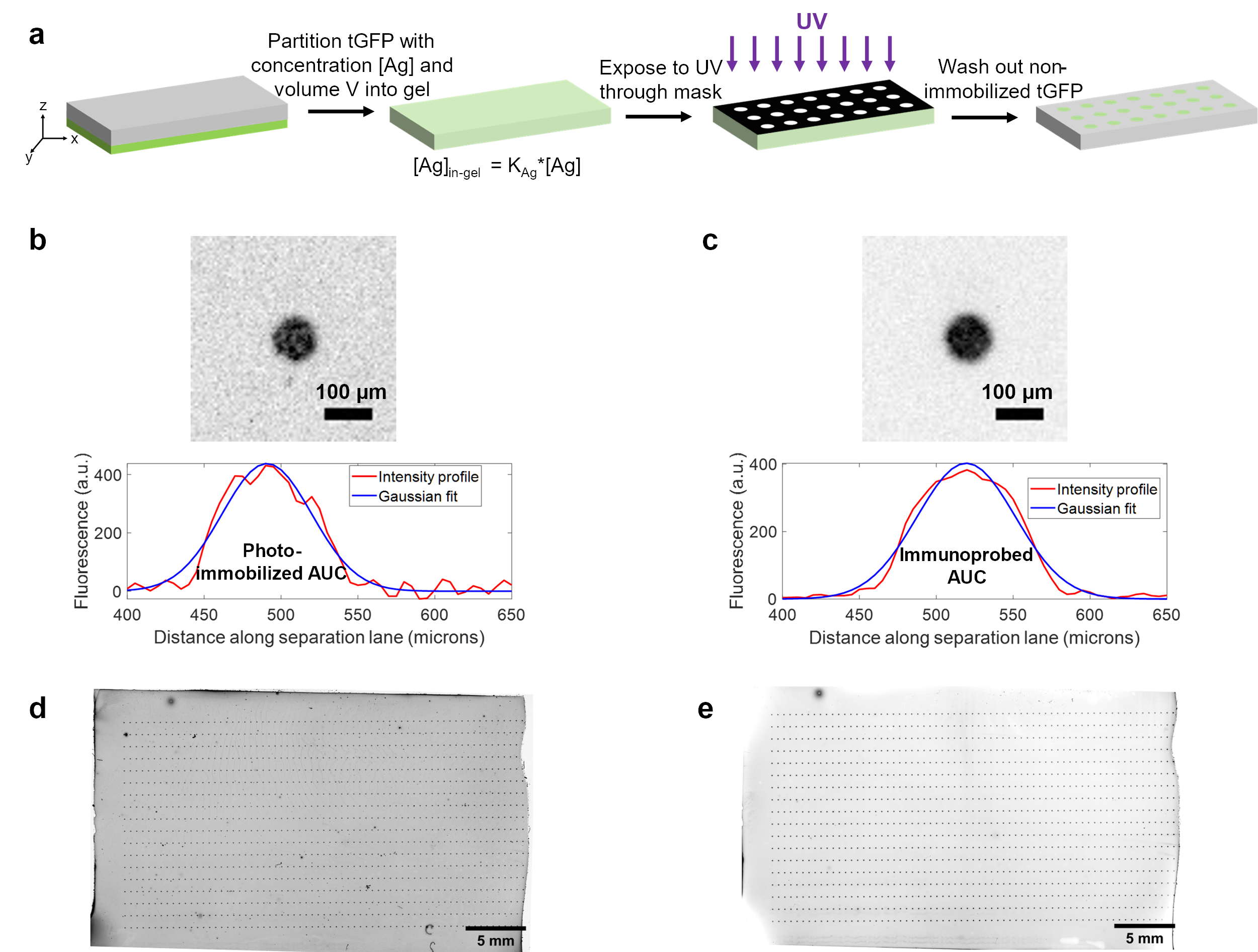


**Figure S2.** Protein photopatterning in polyacrylamide gels. (a) Workflow for generating photopatterned protein spots in a polyacrylamide hydrogel. The number of spots in the schematic is reduced for simplicity. Inverted fluorescence micrographs of a single (b) photo-immobilized Turbo GFP (tGFP) and (c) immunoprobed tGFP spot, with corresponding Gaussian intensity profiles. Inverted fluorescence micrographs demonstrate that ~1100 (d) photo-immobilized and (e) immunoprobed tGFP spots can be patterned and measured on a single array.

**Supplementary Note S1: Control of antibody/antigen stoichiometry and target antigen placement with protein photopatterning.**

To test our hypotheses about η sensitivity to local antibody concentration, we sought to design a test sample which allows control of both antibody/antigen stoichiometry as well as the location, size, and number of target antigen spots. To do so, we combine photoclickable gel chemistry^1^ with UV masking to photo-immobilize target antigens in controlled concentrations and spatial patterns within polyacrylamide gels (see **Supplementary Fig. S2a**). When measuring η, it is important that antibody probe concentration is in excess of antigen concentration to ensure that antibodies are saturating epitope binding sites and depletion regions do not form to add confounding spatial variation in η. Tuning parameters in the incubation and mask design steps controls antibody/antigen stoichiometry. The total number of antigen molecules in the gel can be estimated by **Supplementary Equation (S1)**:

$\text{Antigen molecules in gel}\text{ }\text{=}\text{ }\left[ \text{Ag} \right]\text{*V*}\text{K}_{\text{Ag}}\text{*A*γ}$ (Equation S1)

where [Ag] and V are the concentration and volume of antigen solution the gel is incubated with, respectively, K_Ag_ is the partition coefficient of the antigen in the gel, A is the fractional open area on the photomask, and γ is the photo-immobilization efficiency. A can be easily tuned via mask design, by altering the spacing and size of the UV-exposed areas.

Similarly, the total number of antibody molecules in the gel can be estimated by **Supplementary Equation (S2)**. Because antibodies are distributed across the entire half slide and not photo-immobilized in place, the A and γ terms do not play a role:

$\text{Antibody molecules in gel=}\left[ \text{Ab} \right]\text{*V*}\text{K}_{\text{Ab}}$ (Equation S2)

Here, we have photo-immobilized (see **Supplementary** **Fig. S2b**) and immunoprobed (see **Supplementary** **Fig. S2c**) ~1100 protein spots on a ~22 mm x ~35 mm polyacrylamide gel area (see **Supplementary** **Fig. S2d** and **S2e**), demonstrating the ability to measure η with dense spatial sampling. To ensure antibody was in excess of tGFP for immunoprobing experiments, we calculated the necessary concentration of tGFP and antibody with which to incubate the gels to ensure the antibody/tGFP ratio was >>1. In our system, we determine the antibody/tGFP ratio is 23.5:1, based on a photo-immobilized spot diameter of 100 microns and spacing of dx = 500 microns and dy = 1000 microns, as well as the previously-published^2^ value of γ_tGFP in 8%T_ = 0.275. Partition coefficients of tGFP and antibody were estimated according to **Supplementary** **Note S2**.

**Supplementary** **Note S2: Estimation of partition coefficients.**

The partition coefficient of tGFP in an 8%T polyacrylamide gel was calculated from Ogston’s ideal size-exclusion model^3^, which relates the partition coefficient (K) of a solute to its hydrodynamic radius (R_h_), as well as to the polymer volume fraction (Φ) and polymer fiber radius (a_f_) of the matrix according to **Supplementary** **Equation (S3)**:

$\text{K = exp[-Φ}{\text{(1+}\frac{\text{R}_{\text{h}}}{\text{a}_{\text{f}}}\text{)}}^{\text{2}}\text{]}$ (Equation S3)

Φ can be calculated from the gel density (%T) according to **Supplementary** **Equation (S4)**^4,5^:

$\text{Φ=0.0093 ×\%T-0.03151}$ (Equation S4)

We assume R_h_ of tGFP is similar to the R_h_ of enhanced GFP, which was previously reported^6^ to be 2.4 nm. K of an Alexa Fluor 647-labeled antibody (R_h_ ≈ 6.11 nm) was experimentally measured to be 0.085 in 6%T polyacrylamide gel; using these values and the adjusted Ogston model as previously described^4^, we can back-calculate a_f_ to be 0.647 nm. Thus, we estimate the partition coefficient of tGFP in an 8%T gel to be 0.386.

Because antibodies were diluted in a solution containing 2% wt/vol BSA, the partition coefficient of an unlabeled antibody in an 8%T gel was calculated as 0.0282 using the adjusted Ogston model^7^ to account for the interactions between antibody and BSA. Adjusted Ogston model calculations were performed as previously-reported^4^, except that in the case of this experiment, Φ was calculated for an 8%T gel and R_h_ of an unlabeled antibody^8^ (5.41 nm) was used.

**Supplementary** **Note S3: Impact of higher temperature on antibody probe diffusive timescale.**

To determine whether increasing the temperature of the antibody fluid layer could increase antibody diffusivity enough to sufficiently reduce the timescale of lateral antibody probe diffusion across the fluid layer, we estimated the diffusive timescale of antibody probe across the fluid layer at 37 ^o^C. The diffusivity of antibody probe at 37 ^o^C was calculated to be 6.02 $\times$ 10^-11^ m^2^/s, based on the Stokes-Einstein equation (**Supplementary Equation S5**) and parameters in **Supplementary Table S1**:

$D=\frac{k_{B}T}{6\pi\mu r_{H}}$ (Equation S5)

| **Table S1: Parameters used to estimate antibody probe diffusivity** | | |
| --- | --- | --- |
| **Symbol** | **Name** | **Value** |
| $k_{B}$ | Boltzmann constant | 1.38 $\times$ 10^-23^ J/K |
| T | Temperature | 310 K |
| µ | Dynamic viscosity of water at given temperature^9^ | 6.97 $\times$ 10^-4^ Pa s |
| $r_{H}$ | Hydrodynamic radius of antibody probe^8^ | 5.41 nm |

Using the estimated antibody probe diffusivity at 37 ^o^C and Equation 3 in the main text, we estimate that the timescale of antibody probe diffusion across a 45 mm distance in free solution is 98 days. Thus, increased temperature will not sufficiently enhance antibody diffusion to achieve uniform antibody probe distribution across the sample-fluid layer interface. Raising the temperature may also alter antibody-antigen binding affinity^10^, increase the rate of sample drying, and (at higher temperatures) induce protein denaturation.

**Supplementary Note S4: Estimation of cost of immunoprobing with concentrated probe.**

We estimate the amount and cost of antibody required to ensure [Ab]_sample_ > K_D_ (so that η does not substantially depend on intra-assay variation in antibody probe concentration). Because K_D_ of commercial antibodies is rarely reported but can be as high as µM, we assume that [Ab]_sample_ must be 10 µM to ensure [Ab]_sample_ > K_D_. In the case of single-cell immunoblotting, where target antigens are immobilized within an ~8%T polyacrylamide sieving gel, thermodynamic partitioning limits in-gel antibody concentration. We estimate $\frac{\left[ Ab \right]_{sample}}{\left[ Ab \right]_{solution}}=0.0282$ (see **Supplementary Note S2**), and thus the gel must be incubated with an even higher concentration of antibody probe (354 µM). Each single-cell immunoblot is incubated with 40 µL of probe solution. 40 uL of 354 µM antibody equates to 2.13 mg antibody. Assuming primary antibody costs $4/µg (the approximate cost of primary antibodies used in this study), 2.13 mg primary antibody would cost $8520. Secondary antibodies are generally cheaper (~$0.24/µg for the secondary antibodies used in this study), but would cost an additional $509). Thus, the total cost to probe a single immunoblot with concentrated antibody probe to ensure [Ab]_sample_ > K_D_ would be >$9,000.

**References**

1. Hughes, A. J. & Herr, A. E. Microfluidic Western blotting. *PNAS* **109**, 21450–21455 (2012).

2. Hughes, A. J. *et al.* Single-cell western blotting. *Nature Methods* **11**, 749–755 (2014).

3. Ogston, A. G. The spaces in a uniform random suspension of fibres. *Trans. Faraday Soc.* **54**, 1754–1757 (1958).

4. Su, A., Smith, B. E. & Herr, A. E. In situ measurement of thermodynamic partitioning in open hydrogels. *Anal. Chem.* (2019) doi:10.1021/acs.analchem.9b03582.

5. Baselga, J., Hernández-Fuentes, I., Masegosa, R. M. & Llorente, M. A. Effect of Crosslinker on Swelling and Thermodynamic Properties of Polyacrylamide Gels. *Polymer Journal* **21**, 467–474 (1989).

6. Bhunia, D., Chowdhury, R., Bhattacharyya, K. & Ghosh, S. Fluorescence fluctuation of an antigen–antibody complex: circular dichroism, FCS and smFRET of enhanced GFP and its antibody. *Phys. Chem. Chem. Phys.* **17**, 25250–25259 (2015).

7. Lazzara, M. J., Blankschtein, D. & Deen, W. M. Effects of Multisolute Steric Interactions on Membrane Partition Coefficients. *Journal of Colloid and Interface Science* **226**, 112–122 (2000).

8. Armstrong, J. K., Wenby, R. B., Meiselman, H. J. & Fisher, T. C. The Hydrodynamic Radii of Macromolecules and Their Effect on Red Blood Cell Aggregation. *Biophysical Journal* **87**, 4259–4270 (2004).

9. Appendix C: Physical Properties of Water. in *MWH’s Water Treatment: Principles and Design, Third Edition* 1861–1862 (John Wiley & Sons, Ltd, 2012). doi:10.1002/9781118131473.app3.

10. Reverberi, R. & Reverberi, L. Factors affecting the antigen-antibody reaction. *Blood Transfus* **5**, 227–240 (2007).
